# TRAINING INTERPROFESSIONAL STUDENTS TO LEAD AND COLLABORATE IN THE FIELD OF GERONTOLOGY AND OLDER ADULT CARE

**DOI:** 10.1093/geroni/igad104.2124

**Published:** 2023-12-21

**Authors:** Jennifer Wong, Chun-Ya Belinda Kang

**Affiliations:** Wallis Annenberg GenSpace, Los Angeles, California, United States; Wallis Annenberg GenSpace, Los Angeles, California, United States

## Abstract

With the historic demographic transition of older populations outnumbering younger ones comes an urgent need for services and support for older adults, which entails an integration of various fields. Simultaneously, there has been an alarming decline in the amount of professionals entering older adult care. Furthermore, colleges and universities provide limited education on the service of older adults, and even less fieldwork exposure to older demographics. As such, academia and fieldwork teams must prioritize the training of students in the field of gerontology and of equal importance, students in adjacent fields in order to recruit and equip the incoming workforce in older adult care. Moreover, the training must be integrated and emphasize leadership, as the complex care of older adults requires an interprofessional team engaged in multi-level collaboration. Wallis Annenberg GenSpace is an innovative community space dedicated to older adults and is invested in combating ageism and promoting lifelong learning. GenSpace serves as a fieldwork placement for gerontology, occupational therapy, communication, and public health students. Through supporting community programs and larger policy level work, students are immersed in various aspects of gerontology. Students discover a diversity of crucial ways that their respective professions can contribute to serving older adults. Additionally, GenSpace’s team-based environment allows students to understand the various roles of their peers and prepare to build cross-sectoral partnerships when they enter the workforce. The orientation, curriculum, and outcomes of the student fieldwork completed thus far will be presented.

